# Integration in primary community care networks (PCCNs): examination of governance, clinical, marketing, financial, and information infrastructures in a national demonstration project in Taiwan

**DOI:** 10.1186/1472-6963-7-90

**Published:** 2007-06-19

**Authors:** Blossom Yen-Ju Lin

**Affiliations:** 1 Institute of Health Service Administration, China Medical University, Taiwan

## Abstract

**Background:**

Taiwan's primary community care network (PCCN) demonstration project, funded by the Bureau of National Health Insurance on March 2003, was established to discourage hospital shopping behavior of people and drive the traditional fragmented health care providers into cooperate care models. Between 2003 and 2005, 268 PCCNs were established. This study profiled the individual members in the PCCNs to study the nature and extent to which their network infrastructures have been integrated among the members (clinics and hospitals) within individual PCCNs.

**Methods:**

The thorough questionnaire items, covering the network working infrastructures – governance, clinical, marketing, financial, and information integration in PCCNs, were developed with validity and reliability confirmed. One thousand five hundred and fifty-seven clinics that had belonged to PCCNs for more than one year, based on the 2003–2005 Taiwan Primary Community Care Network List, were surveyed by mail. Nine hundred and twenty-eight clinic members responded to the surveys giving a 59.6 % response rate.

**Results:**

Overall, the PCCNs' members had higher involvement in the governance infrastructure, which was usually viewed as the most important for establishment of core values in PCCNs' organization design and management at the early integration stage. In addition, it found that there existed a higher extent of integration of clinical, marketing, and information infrastructures among the hospital-clinic member relationship than those among clinic members within individual PCCNs. The financial infrastructure was shown the least integrated relative to other functional infrastructures at the early stage of PCCN formation.

**Conclusion:**

There was still room for better integrated partnerships, as evidenced by the great variety of relationships and differences in extent of integration in this study. In addition to provide how the network members have done for their initial work at the early stage of network forming in this study, the detailed surveyed items, the concepts proposed by the managerial and theoretical professionals, could be a guide for those health care providers who have willingness to turn their business into multi-organizations.

## Background

Taiwan's National Health Insurance (NHI) under the control of the Bureau of National Health Insurance (BNHI), was launched in March 1995 to replace its social insurance system that was covering 59% of its population: government employees, labourers, farmers and servicemen [1]. By June 2003 the number of people insured had reached 21,956,729 (99%). There were 17,259 medical providers (92%), including 575 hospitals and 16,684 clinics contracted with the BNHI for serving the enrolled population. The unique phenomenon characterized in Taiwan health care industry different from those in the western countries is the freedom of patients to choose the health care providers they want, no matter what their disease severity is. Furthermore, Taiwan people favor the larger scales of facilities and this fallacy leads to the phenomenon of big-hospital shopping. For example, people choose the medical centers which are accredited as the highest level of medical science in Taiwan when they only suffer from a common cold.

In the spring of 2003, the SARS epidemic viciously attacked the health of Taiwan's people. The people's freedom to choose medical providers caused the national health authority to barely control and traced the flow of epidemic. This event made Taiwan national health authorities rethink what happened and how it damaged under the traditional fragmented health care providers in Taiwan. One health reform launched was named the "Primary Community Care Network (PCCN) demonstration project", a nationwide health care financing program funded by the Bureau of National Health Insurance (BNHI) in March 2003 and it was a new model for the Taiwan government to redefine the role of family physicians in the health care delivery system. A PCCN in Taiwan consists of a group of clinic physicians whose medical jobs are viewed as family care and at least one hospital for secondary or tertiary care. The idea of member component design in PCCNs was aimed to lead the Taiwan citizens to choose one clinic physician as their personal family physician for health maintenance and this family physician also would have the responsibility of referring the patients to specialty care if necessary. From a national health authority perspective, they expected the Taiwan people to put an end to their fallacy that "bigger is better" for health care organizations and establish the idea of "human health", starting with prevention and primary care, followed by secondary or tertiary care, emphasizing health promotion and maintenance instead of disease curing. Furthermore, it could decrease the inappropriateness of medical usage, i.e., over-uses of secondary and tertiary medical services in the high-tech hospitals. In addition, the national health authority was expected to drive the traditional fragmented heath care providers into coordinated medical multidisciplinary teams and share the limited medical resources through the PCCN demonstration project. In summary, the PCCN demonstration project was aimed to: 1) change the traditional patients' customs of freely choosing health care organizations and establish referral channels along the continuum of care, and 2) establish partnerships among the primary care clinics and hospitals to provide a continuum of health care services. It was also expected to establish the primary care system of family physicians to provide whole-people health care and improve care quality [1].

Partnership structures in the PCCNs represent the virtual vertical (i.e., between the member clinics and hospitals) and virtual horizontal (i.e., among the member clinics) aspects of organizing, which designate the formal relationships between individuals and the total network and include organizational design to ensure effective communication, coordination, and integration across the total network. Each PCCN consists of five to ten clinics: half of them should offer the services of general medicine, internal medicine, surgery, obstetrics and gynecology, pediatric, or family medicine. And each PCCN has a central headquarters, usually in one of the clinic facilities, to coordinate and integrate the network. All the clinic physicians in a PCCN are assigned the roles of "family physicians" or "gatekeepers" who recruit people from the local community, keep background and medical files on them, certify family physician education training programs, and hold office hours in the member hospital, where they serve as joint faculty members for further medical consultations or medical utilizations of labs and tests, if necessary. In addition, the hospital member is asked to help clinic physicians in their network to set up a medical information system, share hospital resources (medical equipment and library literature) with the clinic physicians in their network and establish referral channels among the network members. Furthermore, this new demonstration model tries to minimize the barriers to patient access by setting up 24-hour a day, 7-day a week medical consultation telephone lines for providing urgent services onsite and for taking care of the patients whose family physicians' practices are closed to assure seamless care channels. The BNHI funded these extra demonstration actions, at around one hundred thousand US dollars (i.e., NT$3,500,000) for each PCCN under the current fee-for-service payment system [1].

**Figure 1:**
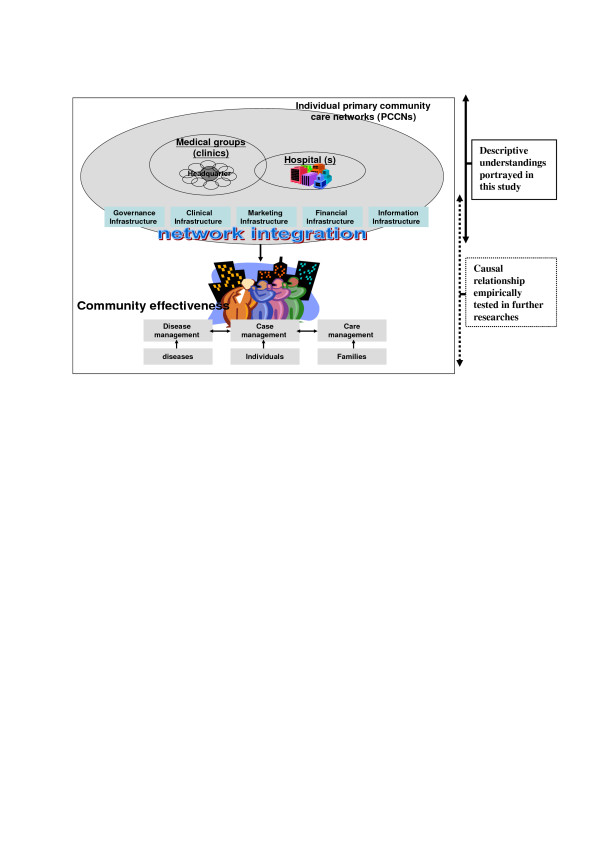
Example of structure and responsibilities of individual PCCNs in Taiwan and conceptual framework of network integration.

Figure 1 describes the organizational structure of individual PCCNs introduced in the demonstration project in Taiwan.

To date, the PCCN demonstration project has been in operation for more than three years. There have been 268 PCCNs formed in the period of 2003 to 2005 around Taiwan. The geographical distributions of PCCNs and their members were described in Table 1. Analyzing all 1,557 participating clinic members in the demonstration project in terms of medical specialties, they cover general medicine, internal medicine, surgeries, obstetrics and gynecology, pediatrics, family medicines, otolaryngology, ophthalmology, rehabilitation medicine, dermatology, and psychiatry, with 237 clinics providing more than two specialties. On the other hand, each PCCN recruits at least one district or regional accredited hospital for acute care demands (required for network members) and a medical center for tertiary care support (not required for network members). There are 6 medical centers, 52 regional hospitals, and 71 district hospitals joining in the demonstration project. See Table 1 for more detailed information about the PCCN members.

To date, there have been few empirical studies of the working relationships that have developed between members of the PCCN program. Partnership needs a method to determine at an early stage, to make sure whether they are making the most of collaboration [2] and the acceptance of the contracting networks in Taiwan as an organizational innovation worthy of greater diffusion deserves to be explored. Therefore, this study used a structured questionnaire to characterize the relationship among the members in the individual PCCNs, with regard to governance, clinical, marketing, financing, as well as information integration infrastructures. The results of this study provide descriptive analyses in detail to map the partnership developments, to enrich the body of knowledge of the partner relationships and to help policy makers understand the coordinated efforts of these health care providers which have developed under this system. It also provides the recommendations for heath policy decision-making and management of networks of health care providers for the future involvement.

**Table 1:** Geographic distributions and medical specialty components of the PCCNs in Taiwan

	**Frequency**		
	
	**PCCNs**	**Clinic members**	**Hospital members**
**Geographical distributions**			
Taipei region	76	435	28
Northern region	44	256	23
Central region	61	390	35
Southern region	37	201	15
Kao-Ping region	44	242	22
Eastern region	6	33	6

**Total**	**268**	**1557**	**129**
**Medical specialty of clinic members**			
General medicine	-	407	-
Internal medicine	-	230	-
Surgery	-	109	-
Obstetrics and gynecology	-	181	-
Pediatric	-	279	-
Family medicine	-	323	-
Otolaryngology	-	155	-
Ophthalmology	-	68	-
Rehabilitation medicine	-	25	-
Dermatology	-	27	-
Psychiatry	-	9	-
**Classification of hospital members**			
Medical centers	-	-	6
Regional hospitals	-	-	52
District hospitals	-	-	71

**Note**: - **: not applicable**

## Methods

This study was aimed at providing descriptive analyses to map the partnership development. To understand the actual integration actions done by network members, the theoretical concept employed by network partnerships were described and then the derived survey instrument was developed.

### Theoretical framework for organization design of network integration

The rapid organizational changes in the health care industry have driven theorists from every discipline and across the world to seek an approach that allows organizations to flourish. Organization theory allows investigators to profile an organization from the aspect of patterns and regularities in organizational design and behavior. In the early 20^th ^century, classical management theorists claimed that an organization has "a best way" to be organized and managed [3]. That implied that all organizations would own the "same" organizational styles or structures. In the 1960s, several theorists [4-8] challenged this assumption by applying a "contingency approach" to propose that there is no best way to organize an organization, and that the effectiveness of an organizational structure varies with the situation of an organization. Furthermore, it is proposed that the best way to organize an organization depends on the nature of the environment to which the organization relates.

Contingency theory delineates the concepts "organization's internal features," "the demands of organizational environments," "best adaptation," and, the most important and difficult of all, "best match" [9]. Lawrence & Lorsch [7] argued that environments characterized by uncertainty and rapid rates of change in market conditions or technology impose different demands, including constraints and opportunities, on organizations than do placid and stable environments. Similarly to Lawrence and Lorsch's views mentioned above, Galbraith [10,11] stressed the contingency perspective on information processing. The information-processing approach emphasizes that environment, size, and technology impose different information-processing requirements on organizations, and thus an organization must be designed to encourage information flow in both vertical and horizontal directions to achieve the overall tasks of the organization and, finally, organizational effectiveness [11-14].

Some theorists have criticized conventional contingency theorists who presume that organizational structure is driven by the environment. Child [15], Miller [16], Van de Ven and Drazin [17], and Tushman and Romanelli [18] raised such criticisms; they argued that organizations become what they are not only because of the environment, but also because of choices made by members, especially choices about strategy and organizational design. As Thompson's words in the book *Organizations in Action *[8] put it, "organizations are not determined simply by their environments (p.27)." He also pointed out that "administration may innovate on any or all of the necessary dimensions, but only to the extent that innovations are acceptable to those on whom the organization can and must depend." Instead of assuming that administrators are highly constrained in their decisions, strategic contingency theorists emphasized "the importance of choice," that is, "the freedom of agency" [15]. Furthermore, Pfeffer [19] explicitly pointed out that "organizational structures are the outcomes of political contests within organizations (p.38)."

Daft [14] proposed a top management model to delineate how "a strategy is a plan for interacting with the competitive environment to achieve organizational goals." He stated that the major responsibility of top management is to determine the goals, strategy, and design of an organization to adapt to a changing environment. To assess the external and internal environments of an organization seems to be the first task for top managers in defining an organization's goals and missions. Then, guided by the goals and missions of the organization, top managers shape the design of the organization, including structural forms, information system, technology, human resources, organizational culture, and inter-organizational linkages, to achieve the final organizational performance.

Integration refers to the mechanisms of coordination, the ways guided to partnership goals to fit internal and external conditions [7,20,21]. In the early 1990s, proposals for US national health care reform recognized the need for integrating mechanisms to achieve both financial success and quality of care of a well-organized system of care [22,23]. Several researchers also viewed inter-organizational cooperation as resource exchanges, including client referrals, money, and staff [24-27]. From practical ways of viewing integration, the success of integration lies in the coordinative mechanisms and partnership working that support it [28], including an administrative organization that coordinates the operations of various health care services; a management information system that integrates clinical, utilization, and financial data and follows clients across different settings; a care coordination program such as case management or disease management that works with clients to arrange health care services; and a financial mechanism that enables pooling of funds across services [29-35]. Fox [36] suggested the success of integrated health networks should ensure that the new business link such aspects as technology, functional skills, customer access, management, or products that can be shared across both the core and the new business; to conduct market financial evaluation; to share the risk of vertical integration with outside entities, to develop the management structure that can reflect the degree of coordination necessary to support the core business activities; to ensure that the integration strategy meets the needs of customers, including medical treatment, the use of medical technology, and the preferred methods of purchase; and to measure the new business by its value to the enterprise as a whole, rather than by its profitability as a stand-alone entity.

In summary, the effects that integration in inter-organizational designs has on network management were substantial from a managerial perspective. Borrowing the ideas of strategic contingency perspective [8,15,19] and top management model [14], it could be imply that success (organization performance) in reengineering a network lies in the integration of process and services (see Figure 1), including leadership/governing structure, teamwork between disciplines and patient care, financial planning, and information systems, characterized as the constructs of *governance*, *clinical*, *financial*, and *information *infrastructures, respectively, in this study. In addition, another construct, *marketing *infrastructure, was especially important and designed to explore for PCCNs in this study because of patients' freedom of making healthcare choice and the traditional fragmented health care systems by individual health care organizations in Taiwan. One major reason for Taiwan people's hospital shopping preferences was that Taiwan people usually believe the bigger the facility, the better capacities a facility has no matter on any aspect from medical professionals to tangible medical equipment and plants. And this fallacy made the public want to overuse the facility with high-tech medical services no matter if it fits their needs. From the health policy and management perspectives, therefore, the health care providers were encouraged to market their services as a new corporate identity and brand strategy [37], including offering tangible resources such as books, libraries, medical equipment, and intangible resources such as knowledge and information exchanges (education) and reputation sharing one another among PCCN members. Furthermore, through the process of marketing resource exchanges, therefore, each PCCN could establish the images of "one system, one brand and quality" for the public and for the health care providers. It also makes it be more visible to the public.

The five integration infrastructures of network management were constructed as a conceptual framework in this study to help to portray how the PCCN members have done. The survey instrument development was described in the following.

### Survey instrument development: integration infrastructures and measurements of partnerships

Based on the five integration infrastructures of network management, the structured questionnaire were derived from extensive literature reviews.

#### Governance infrastructure

Governance assumes the broad responsibility for organizational goals and survival and involves the series process of setting and monitoring organizational goals and strategy development through a board of representatives [38]. Governance or administrative integration infrastructure in establishing network partnerships refers to administrative structures (or responsibilities) created to facilitate communication, clear lines of authority, accountability, and responsibility for patient care services; to negotiate budgets and financial trade-offs; and to present a cohesive, consistent message in interactions with external agencies and the community [29,39-41] and most important for members in contract agreements, to manage participation [33]. From a multidisciplinary perspective, Mitchell and Shortell [42] applied the concepts of governance and management characteristics in effective community health partnerships. The construct of governance involved several tasks, including setting priorities for strategic goals, choosing the membership composition, obtaining the necessary financial resources, and setting up the accountability systems, and so on. The construct of the management refers to the tasks of engaging and maintaining organizational members' interest in a shared vision and mission, providing appropriate structures and coordination mechanisms for the specified strategies, promoting constructive conflicts and managing destructive conflicts, implementing information systems to monitor the dynamics, adjusting the leadership in the overall membership, and so on. The issues of governance and administrative integration in the PCCNs could include [2,38,40,41,43-47]:

• planning the shared visions and missions

• determining the shared service strategies, cooperation priorities, policies and principles

• identifying the information needed and how to get it

• organizing the network dynamics and member roles

• leading and managing the conflicts and communication

• designing and controlling the shared network performance systems, including indicator settings, feedbacks, and accountability.

#### Clinical infrastructure

The idea of care integration begins through such public programs that include social workers in public welfare departments, caseworkers in mental health, or nurses in public health departments. In the late 1980s, care integration was deemed necessary for the streamlining of care and negotiating the maze of long-term care services. At that time, it was referred to as service coordination or case management, or in other related terms [29]. The purpose of care integration is to work directly with patients and their families over time to help them arrange and manage the complex resources that patients may need to maintain health and independent functioning. At the same time, care integration is used to achieve the most cost-effective use possible of scarce resources, by steering patients to the health, social, and support services most appropriate for them at a given time [29]. Conrad and Dowling [33] pointed out that to coordinate and integrate patient care relies on connecting patient services at the different stages of the patient care processes. Care coordination in integrated networks can be achieved through integration of training programs and some clinical services, provision of complementary clinical capabilities, clinical geographic proximity design, clear role definition of each institution, commitment and flexibility of leaderships and medical staffs, and the support of a large referring physician groups embracing the affiliation concepts [48]. The issues of clinical integration in the PCCNs could include [48-50]:

• planning and differentiating target markets based on the clinical services of the network members

• uniting individual clinical professionals for clinical project planning

• designing patient-centered care or case management teams

• establishing committees responsible for patient-centered case report meetings, case referral, transfer, and tracing, file management (record and information exchanges), clinical quality management (quality assurance, improvement, risk and malpractice management, and utilization review), and medical continuing education and on-job education.

#### Marketing infrastructure

Marketing integration refers to how to work together as a whole both from the provider and patient perspectives. One of the case reports interviewing developing integrated delivery system or networks realized that the most important thing is how an integrated system or network is promoted and what is promoted for the consumers [51], including focusing on product development, making sure the branding holds together, marketing directly to consumers, demonstrating values to consumers, and even conducting marketing research to make efforts for the long term. In a health care network with several organizational members and target patients, the marketing infrastructure in PCCNs here refers to provider members' marketing, meaning the resource sharing and market development in a PCCN as a whole. The issues of the marketing integration in the PCCNs could include [37,52-54]:

• sharing the literature and facility publications among the network members

•uniting public promotions such as united activities, electronic and paper media for enhancing the network reputation as "one system, one brand and quality"

• differentiating target markets of the network for competing in the medical industry.

#### Financial infrastructure

Comprehensive, flexible, and adequate financing is a goal of the ideal continuum of care. That component is the most critical and challenging to manage under the changes in the health care delivery environment. Gillies et al. [30] suggested that integrating financial management across operating units adds the greatest value to systems or organizations. In one case study, Bramson et al. [55] also showed that reducing costs through joint purchasing by the radiology departments of a vertically integrated health system could yield substantial savings. The issues of the financial integration in the PCCNs could include:

• budgeting

• uniting equipment, medical materials, and drug purchasing and routine administrative stuff management

• pooling recruitment funds

• designing a financial risk and sharing mechanism.

#### Information infrastructure

Information is an essential component of an organization. A complete information system can help an organization to integrate its individual units and efficiently manage the continuum. The ideal information system for a continuum of care was conceived of and formed in the mid-1980s [56]. During the late 1980s, computer technology began to make an information system feasible and affordable through new computer chips with expanded capability and networking technology. In the 1990s, the individual services of the continuum upgraded their information systems to combine clinical, financial, and utilization data [29]. Some studies have argued that the quality of information systems can drive costs down, because a good information system can give physicians easy electronic access to complete the documentation of the patients' clinical records, better inform them about reimbursement and capitation issues, help them easily associate and manage cases together, and achieve a higher level of professional satisfaction [57,58]. Using Inova Health System, an integrated delivery system in northern Virginia, as an example, Wager, Heda, and Austin [59] showed that by developing a health information network within an integrated delivery system, Inova can have a clinical transaction system for hospitals and other entities, a data repository for decision support and outcome management, a managed care information system to support managed care and capitation contracts, and greater capability to acquire physicians. The issues of information coordination include [60-68]:

• establishing an electronic medical record system, regional information network for patient clinical and administrative data, clinical service arrangements and administrative work

• uniting the system information management and web pages.

The structured questionnaire was developed with the wording of practical managerial actions based on the five concepts just mentioned. There were 19 survey items on governance infrastructure, 25 on clinical infrastructure, 13 on marketing infrastructure, 20 on financial infrastructure, and 7 on information infrastructure. All 84 items were, simultaneously, applied to examine the relationships of the clinic's peer members and the relationship of clinic and hospital members in a PCCN, and it resulted in a total of 168 survey questions. The detailed information of the item questions was listed in Table 2, 3, 4, 5, 6. The structured questionnaires were drafted from previous literatures and then examined by two academic professors for theoretical accuracy. Then one pilot study was pre-tested for the PCCN pioneers (i.e., 92 network clinic members) and 116 hospital providers which have partner relationships with other health care organizations (i.e., hospitals, clinics, long-term care facilities). The wordings and meanings of each question item were revised to assure content validity. The Cronbach α values for the five integration constructs – governance, clinical, marketing, finance, and information infrastructure were 0.946, 0.958, 0.932, 0.944, and 0.898 for the measures of clinic-clinic member relationships; and 0.945, 0.949, 0.916, 0.948, and 0.896 for the measures of clinic-hospital member relationships.

**Table 2:** Item descriptions and analyses for integration dimension: governance infrastructure (n = 928)

**Item Descriptions**		**Clinic-clinic** **Relationship (1)**			**Clinic-hospital** **Relationship (2)**			
				
		**Frequency (%)**			**Frequency (%)**			**Pair-t tests**
				
		**Disagree**	**Fair**	**Agree**	**Disagree**	**Fair**	**Agree**	
**1**	Obey the determined deals	0.97	10.34	88.69	1.62	11.85	86.53	(1)>(2)**
**2**	Control the network plans and goal achievements	12.50	29.63	57.87	16.59	31.79	51.62	(1)>(2)***
**3**	Design and employ the network performance indicators	21.23	31.47	47.31	17.67	30.06	52.26	(1)<(2)***
**4**	Timely performance feedbacks to network members	4.53	24.46	71.01	4.53	23.81	71.66	
**5**	Regulate the availability of patient data in the network	2.91	21.66	75.43	2.37	23.92	73.71	
**6**	Determine the distribution principals of gaining	7.22	32.11	60.67	6.47	32.33	61.21	
**7**	Determine cooperation policy and principals	8.41	31.03	60.56	7.87	31.25	60.88	
**8**	Determine disintegration policy and principals	7.87	31.47	60.67	6.68	30.93	62.39	(1)<(2)*
**9**	Determine conflict resolution models	7.22	28.99	63.79	6.68	29.20	64.12	
**10**	Communicate business strategies among network members	5.28	27.69	67.03	4.74	27.69	67.03	
**11**	Establish fair coordination mechanism	27.91	38.04	34.05	26.19	38.04	35.78	(1)<(2)**
**12**	Establish communication models and channels	19.94	37.07	43.00	18.75	37.93	43.32	
**13**	Understand the roles of network members	7.22	34.38	58.41	7.33	34.48	58.19	
**14**	Take care of all members' benefits on strategic planning	5.17	28.02	66.81	4.96	27.26	67.78	
**15**	Determine the united principals for individual members' development	1.40	19.18	79.42	1.94	18.97	79.09	
**16**	Understand the members' goals and strategies	2.05	18.21	79.74	2.05	19.07	78.88	
**17**	Compatible goals and strategies for all members	3.13	20.91	75.97	3.56	21.98	74.46	
**18**	Invest in sufficient inputs for the network development (goals and strategies)	7.33	30.17	62.50	6.57	30.71	62.72	
**19**	Establish coordination mechanisms for the whole network and individual development	4.20	25.00	70.80	4.63	25.75	69.61	

**Note**:

1. Paired t test: *p < 0.05; **p < 0.01; ***p < 0.001

2. Items measured originally as Likert scale 1 (strongly disagree)-5 (strongly agree); they were recoded as disagree (Likert score 1 and 2), fair (Likert score 3), and agree (Likert score 4 and 5) in frequency counts

**Table 3:** Item descriptions and analyses for integration dimension: clinical infrastructure (n = 928)

**Item Descriptions**		**Clinic-clinic** **Relationship (1)**			**Clinic-hospital** **Relationship (2)**			
				
		**Frequency (%)**			**Frequency (%)**			**Pair-t tests**
				
		**never thinking**	**brain storming**	**acting**	**never thinking**	**brain storming**	**acting**	
**20**	Plan and differentiate market areas based on the clinical services of the network members	45.80	21.77	32.44	43.86	21.66	34.48	(1)<(2)**
**21**	Unite individual clinical professionals to plan the certain projects	34.59	34.16	31.25	33.30	32.76	33.94	(1)<(2)*
**22**	Design the patient-centered case management teams	44.40	27.59	28.02	40.63	28.77	30.60	(1)<(2)***
**23**	Hold the patient-centered case report meetings	30.93	23.81	45.26	24.46	24.78	50.75	(1)<(2)***
**24**	Establish the committee responsible for case referral, transfer, and tracing	16.59	17.46	65.95	7.54	14.66	77.80	(1)<(2)***
**25**	Establish the committee responsible for file management (record and information exchanges)	19.18	20.04	60.78	9.70	17.13	73.17	(1)<(2)***
**26**	Coordinate clinical services within the network	17.13	20.91	61.96	10.56	19.29	70.15	(1)<(2)***
**27**	Redesign the clinical services to avoid the redundancy	33.73	22.41	43.86	28.77	21.88	49.35	(1)<(2)***
**28**	Appropriately share clinical resources within the network	15.52	21.23	63.25	8.51	18.64	72.84	(1)<(2)***
**29**	Appropriately integrate the clinical services of network members to achieve cost effectiveness of patient care	23.06	19.61	57.33	14.22	18.64	67.13	(1)<(2)***
**30**	Establish and share the experience of quality assurance and improvements	18.21	21.23	60.56	11.85	19.61	68.53	(1)<(2)***
**31**	Establish two-direction communication channels for securing clinical quality	19.83	21.66	58.51	11.53	18.53	69.94	(1)<(2)***
**32**	Integrate the activities of quality assurance, quality improvement, risk management, and utilization review	25.22	25.86	48.92	18.00	22.31	59.70	(1)<(2)***
**33**	Establish the policy and principals of quality assurance and improvements	25.11	29.20	45.69	18.00	25.86	56.14	(1)<(2)***
**34**	Unite medical continuing education and on-job education	15.73	14.01	70.26	5.93	10.88	83.19	(1)<(2)***
**35**	Establish patient information of referrals	9.59	14.66	75.75	3.23	10.45	86.31	(1)<(2)***
**36**	Design clinical guidelines	25.00	20.91	54.09	17.24	19.18	63.58	(1)<(2)***
**37**	Design two-directed patient referral systems	10.13	12.93	76.94	4.31	10.24	85.45	(1)<(2)***
**38**	Establish lab/exam referral systems	20.80	18.00	61.21	7.54	13.69	78.77	(1)<(2)***
**39**	Integrate medical records to decrease unnecessary medicine, test, and labs	28.99	25.22	45.80	20.91	25.43	53.66	(1)<(2)***
**40**	Hold quality relevant symposium	19.94	17.56	62.50	13.47	17.13	69.40	(1)<(2)***
**41**	Establish quality indicators	19.40	20.47	60.13	16.16	18.86	64.98	(1)<(2)***
**42**	Establish the reasonable values or thresholds for the designed quality indicators	20.80	22.09	57.11	17.03	21.55	61.42	(1)<(2)***
**43**	Routinely monitor and analyze quality indicators	22.74	22.95	54.31	18.86	22.09	59.05	(1)<(2)***
**44**	Establish committees to deal with medical malpractice	48.38	21.66	29.96	44.72	22.09	33.19	(1)<(2)***

**Note**:

1. Paired t test: *p < 0.05; **p < 0.01; ***p < 0.001

2. Items measured as scale 0 (never thinking), 1 (brain storming), 2 (developing), and 3 (completely acting). And for frequency counts, "acting" was counted by adding the items "developing" and "completely acting" together.

**Table 4:** Item descriptions and analyses for integration dimension: marketing infrastructure (n = 928)

**Item Descriptions**		**Clinic-clinic** **Relationship (1)**			**Clinic-hospital** **Relationship (2)**			
			
		**Frequency (%)**			**Frequency (%)**			**Pair-t tests**
				
		**never thinking**	**brain storming**	**acting**	**never thinking**	**brain storming**	**acting**	
**45**	Share available professional literature and books	42.13	20.26	37.61	20.37	16.92	62.72	(1)<(2)***
**46**	Regularly or irregularly share individual facility reports for updated services	19.94	16.06	64.01	12.50	12.61	74.89	(1)<(2)***
**47**	Release network information for the public through electronic and paper media	25.11	21.88	53.02	14.76	16.49	68.75	(1)<(2)***
**48**	Regularly or irregularly share individual facility reports within the network	34.70	21.12	44.18	15.84	15.73	68.43	(1)<(2)***
**49**	Share individual facility reports within the network	43.75	22.41	33.84	21.01	16.59	62.39	(1)<(2)***
**50**	Unite the publications for the network communication	39.98	23.38	36.64	27.48	20.91	51.62	(1)<(2)***
**51**	Invite the members one others for individual member facility activities	21.66	16.92	61.42	10.45	14.01	75.54	(1)<(2)***
**52**	Cooperate large and small research projects	31.68	21.77	46.55	25.00	19.18	55.82	(1)<(2)***
**53**	Unite social activities to enhance the network reputation	17.35	18.21	64.44	11.75	14.87	73.38	(1)<(2)***
**54**	Release information of medical services of network members to the public to enhance the network reputation	17.67	20.91	61.42	11.96	16.81	71.23	(1)<(2)***
**55**	Identify target markets of the network for health and medical services	25.32	24.03	50.65	19.29	22.52	58.19	(1)<(2)***
**56**	Identify target markets of the network for community health educations	19.40	23.92	56.68	14.44	20.37	65.19	(1)<(2)***
**57**	Identify and develop target markets of the network for competing in the medical industry	30.06	23.92	46.01	24.57	21.34	54.09	(1)<(2)***

**Note**:

1. Paired t test: ***p < 0.001

2. Items measured as scale 0 (never thinking), 1 (brain storming), 2 (developing), and 3 (completely acting). And for frequency counts, "acting" was counted by adding the items "developing" and "completely acting" together.

**Table 5:** Item descriptions and analyses for integration dimension: financial infrastructure (n = 928)

**Item Descriptions**		**Clinic-clinic** **Relationship (1)**			**Clinic-hospital** **Relationship (2)**			
			
		**Frequency (%)**			**Frequency (%)**			**Pair-t tests**
				
		**never thinking**	**brain storming**	**acting**	**never thinking**	**brain storming**	**acting**	
**58**	Unite budget planning	44.61	14.66	40.73	48.81	12.82	38.36	(1)>(2)***
**59**	Unite recruiting funding	64.87	12.61	22.52	67.24	12.28	20.47	(1)>(2)***
**60**	Unite equipment purchasing	76.19	12.61	11.21	77.80	10.99	11.21	
**61**	Unite equipment outsourcing	77.91	12.07	10.02	79.09	11.10	9.81	
**62**	Unite equipment maintenance	74.35	14.12	11.53	73.49	13.25	13.25	(1)<(2)*
**63**	Unite medical materials and drugs purchasing	64.87	20.26	14.87	72.31	16.27	11.42	(1)>(2)***
**64**	Unite medical discard materials dealing	70.91	12.82	16.27	75.97	10.99	13.04	(1)>(2)***
**65**	Unite resources materials dealing	72.84	15.52	11.64	76.19	12.72	11.10	(1)>(2)***
**66**	Unite housekeeping	79.20	12.28	8.51	80.82	10.78	8.41	
**67**	Unite equipment maintenance	77.91	12.39	9.70	78.88	10.88	10.24	
**68**	Share places, materials, equipment	49.57	18.97	31.25	42.35	17.35	40.30	(1)<(2)***
**69**	Unite update equipment and reinvestment	78.13	12.18	9.70	79.31	10.78	9.91	
**70**	Design financial risk and sharing mechanism	66.38	14.12	19.50	66.81	13.79	19.40	
**71**	Unite recruiting fund for the certain services	56.90	17.24	25.86	59.27	16.81	23.92	
**72**	Unite budgeting for the certain services	48.71	17.78	33.51	52.59	17.13	30.28	(1)>(2)***
**73**	Unite professionals to plan the certain project	49.35	20.91	29.74	50.43	19.29	30.28	
**74**	Centralize revenue and earnings for feedback to individual members	54.74	16.81	28.45	59.91	14.98	25.11	(1)>(2)***
**75**	Centralize cask management	59.59	13.47	26.94	64.55	12.61	22.84	(1)>(2)***
**76**	Unite project assessment for investing in new services	59.91	17.13	22.95	61.31	16.92	21.77	
**77**	Design resource distribution principals based on the whole goals	46.88	18.64	34.48	49.14	17.56	33.30	(1)>(2)*

**Note**:

1. Paired t test: *p < 0.05; ***p < 0.001

2. Items measured as scale 0 (never thinking), 1 (brain storming), 2 (developing), and 3 (completely acting). And for frequency counts, "acting" was counted by adding the items "developing" and "completely acting" together.

**Table 6:** Item descriptions and analyses for integration dimension: information infrastructure (n = 928)

**Item Descriptions**		**Clinic-clinic** **Relationship (1)**			**Clinic-hospital** **Relationship (2)**			
			
		**Frequency (%)**			**Frequency (%)**			**Pair-t tests**
				
		**never thinking**	**brain storming**	**acting**	**never thinking**	**brain storming**	**acting**	
**78**	Establish electronic medical record system	23.81	21.44	54.74	17.78	21.66	60.56	(1)<(2)***
**79**	Establish regional information network for pt clinical data	29.85	24.68	45.47	20.26	24.35	55.39	(1)<(2)***
**80**	Establish regional information network for clinical service arrangements	30.60	27.59	41.81	21.88	26.62	51.51	(1)<(2)***
**81**	Establish regional information network for pt administrative data	40.63	27.16	32.22	33.94	26.83	39.22	(1)<(2)***
**82**	Establish regional information network for administrative works, such as registration, billings, and so on	59.38	25.00	15.63	53.45	25.43	21.12	(1)<(2)***
**83**	Establish regional information network for information management	46.12	28.13	25.75	39.66	27.37	32.97	(1)<(2)***
**84**	Establish united web pages	42.35	28.02	29.63	37.72	26.83	35.45	(1)<(2)***

**Note**:

1. Paired t test: ***p < 0.001

2. Items measured as scale 0 (never thinking), 1 (brain storming), 2 (developing), and 3 (completely acting). And for frequency counts, "acting" was counted by adding the items "developing" and "completely acting" together.

### Study subjects

To find the member partnership, we sent questionnaires to 1,557 individual clinics which had belonged to PCCNs for at least one year, based on information contained in the Taiwan Primary Community Care Network List (Bureau of National Health Insurance 2003, 2004 and 2005).

We let clinic members in all PCCNs point out how they coordinate with their peer clinic members and hospital members within a PCCN because individual clinic members could be better informants than hospital members, which need to deal with multiple clinic relationships and therefore might find it hard to describe the coordination involvement one by one with clinic members. Moreover, networks form for various reasons and it might lead to the various involvements by individual network members (i.e., hospital and clinic members). Therefore, using the participating clinics as individual survey units, the results could portray the overall dynamics and processes more authentically and detailed throughout all PCCNs in the demonstration project.

Nine hundred and twenty-eight clinics responded (59.6 %), with 239 clinics in the Taipei region, 165 in the northern region, 241 in the central region, 108 in the southern region, 150 in the Kao-Ping region, and 15 in the eastern region of Taiwan. Ten clinics had not mentioned their practicing locations. There is no statistically significant difference in geographical distribution between the respondents and the study population (χ^2 ^= 4.208, p > 0.05).

### Analytical techniques

The data was first analyzed descriptively with frequency counts (percentage) for each survey item, instead of using mean as a statistical method, because the variation among the respondents may not represent the normal distribution and it might ignore the extreme values for the respondents' answers. To compare how the respondents perceived the strength of integration existing in clinic-clinic and clinic-hospital relationships, paired t-tests were performed for individual survey items, using the original numerical scores.

## Results

### Profiling the partnerships in Taiwan PCCNs: governance infrastructure

With regard to the governance infrastructures, the frequency was counted for each survey item with recalculated scales: disagree (Likert scale 1 and 2), fair (Likert scale 3), and agree (Likert scale 4 and 5) with individual items. In clinic-clinic relationship (Table 2), the majority of clinic members agree that the determined deals were obeyed (Table 2, item 1: 88.69%), the goals and strategies of members were well-understood (Table 2, item 16: 79.74%), and the united principals for individual members were developed (Table 2, item 15: 79.42%). The higher percentages were also found in clinic-hospital relationship in the same items (Table 2). On the other hand, establishing fair coordination mechanism (Table 2, item 11: 27.91%), designing and employing the network performance indicators (Table 2, item 3: 21.23%), and establishing communication models and channels (Table 2, item 12: 19.94%) still occupied higher percentages not developed and deserved to been made the focus of more efforts in the future. Paired t-test analyses for all individual survey items of governance infrastructure showed that the deals obeyed (Table 2, item1) and plans and goals controlled (Table 2, item 2) were achieved more in clinic-clinic relationships than those in clinic-hospital relationships; however, the design of network performance indicators (Table 2, item 3), development of disintegration policy and principals (Table 2, item 8), and the establishment of fair coordination mechanism (Table 2, item 11) were reached more in clinic-hospital relationships than those in clinic-clinic relationships.

### Profiling the partnerships in Taiwan PCCNs: clinical infrastructure

Examining the extent of clinical infrastructure for network members, establishing two-directed patient referral systems and patient referral information files (Table 3, items 35 & 37) and uniting medical continuing education and on-job education (Table 3, item 34) were shown at a highly implemented rate in clinic-clinic (more than 70%) and clinic-hospital (more than 80%) relationships. On the other hand, network members had higher percentages (more than 40%) not to think about the possible integration mechanisms including establishing committees to deal with medical malpractice (Table 3, item 44), planning and differentiating clinical market areas (Table 3, item 20), and designing patient-centered case management teams (Table 3, item 22). Overall, there was better clinical integration involvement for all the described items in clinic-hospital relationships than those in clinic-clinic relationships within a network in this study (see Table 3, paired t-tests, p < 0.05).

### Profiling the partnerships in Taiwan PCCNs: marketing infrastructure

For marketing planning, the clinics had better integrated marketing activities with their respective hospitals than with peer clinic members within PCCNs for all studied items (Table 4, paired t-test, p < 0.05). Examining the clinic-clinic relationships, uniting social activities (Table 4, item 53), sharing the individual facility reports for updated services (Table 4, item 46), public promotion (Table 4, item 54), and uniting and joining the facility activities (Table 4, item 51) were the top four marketing works done among clinic members (more than 60% implemented rate); and those items also showed a higher implemented rate (more than 70%) between clinic and hospital members.

On the other hand, facility assets such as reports (Table 4, item 49), and professional literatures and books (Table 4, item 45) were not well-shared among clinic members ("never-thinking" rate: 42.13%). In addition, uniting the network publication could make more efforts in the future ("never-thinking" rate in item 50: 39.98%). The room for clinic-hospital partnership to think about acting was kind of different from those in the clinic-clinic relationship. In addition to the uniting publication that can be encouraged to improve the clinic-hospital relationship (Table 4, item 50: 27.48%), cooperating in research projects (Table 4, item 52: 25.00%) and identifying and differentiating target markets (Table 4, item 57: 24.57%) had still more opportunities to be focused on in the future.

### Profiling the partnerships in Taiwan PCCNs: financial infrastructure

The PCCN members were found to have a lower extent of financial integration as evidenced by higher percentage of ''never thinking'' scale about the survey items on almost all items (see Table 5). Slightly more integration (that is, ''acting'' rate) was found in only four items both in clinic-clinic relationships and in clinic-hospital relationships, including uniting budget planning (Table 5: item 58), sharing places, materials, and equipment (Table 5: item 68), uniting budgeting for certain services (Table 5: items 72), and designing the resource distribution principals based on the whole network goals (Table 5: item 77).

Further examining the financial infrastructure in clinic-clinic relationship and clinic-hospital relationship, paired-t tests revealed that clinic-hospital partnerships were involved more in places, materials, and equipment sharing and maintenance (Table 5, items 62 and 68) (p < 0.05) and higher financial infrastructure coordination exists in clinic-clinic relationships (Table 5, items 58, 59, 63–65, 72, 74, 75, and 77) (p < 0.05).

### Profiling the partnerships in Taiwan PCCNs: information infrastructure

There was significantly greater integration of information in clinic-hospital than clinic-clinic relationships in all items in this category (Table 6, paired t-tests, p < 0.001). The greatest integration was found in electronic patient records (Table 6: item 78), followed by information integration for patient data (Table 6: item 79) and clinical service arrangements (Table 6: item 81). The lowest level of integration in information infrastructure was found in administrative works such as registration, billing and so on (Table 6: item 82, ''never-thinking'' rate more than 50%) within network members.

## Discussion

In this study, we surveyed 943 clinics that had belonged to Taiwan PCCNs for more than a year to understand the nature and extent of integration to which they and their associated PCCN members (clinics and hospitals) had in governance, clinical, marketing, financial, and information infrastructures. It was found a wide variance in the kind and degree of integration among them and a lot of room for better integration (Table 2, 3, 4, 5, 6).

From the governance perspective, we found lower integration was found in the establishment of fair coordination mechanism (Table 2: item 11) among member clinics and member hospitals. Coordination could be viewed from different perspectives, including the use of standardized languages and forms, organizational rules and procedures, the establishment of common rules, policies, and procedures, and the monitoring through memos, reports, and a computerized information system [69,70]. Facing the cumbersome integration processes, it suggests that each PCCN's headquarters should become actively involved and clarify the authority, responsibility and accountability of individual members, identify the potential conflict sources, and publicize the rules and regulation of network integration dynamics covering decision making processes, market planning, clinical teamwork designs, and financial reports of individual network members. These actions could enhance the trust and respect of network members one another and could improve the small extent of integration found in this study about the mechanisms for communication models and channels in the PCCNs (Table 2: item 12). From the network management perspective, communication could occur between the various entities such as between hospital and clinics, primary care physicians and specialists, managers and clinical professionals, and even among the clinical professionals in the network. To develop effective and timely communication channels was the key for the management of integrated organizations [30-32] and could alleviate the tensions that sometimes occur in the dynamics of the multi-organizations. In this study, it was found a low level of involvement of medical teams in medical projects, patient-centered case management, and case report meetings among the network members (Table 3: item 21, 22, and 23) from a clinical integration perspective. Several researchers have addressed that clinical integration providing a process of medical management, care management, case management, and patient management designed to transform the traditionally fragmented delivery system into a more cohesive system [71], and lead to higher service quality and assure financial objectives [72,73]. More attention could be paid to these activities in the future. In addition, it was also found less integration in planning and differentiating clinical market areas (Table 3, item 20) among the network members in the category of clinical infrastructure. This may result from the existing specialty diversities in individual PCCNs, which might not need to involve planning and differentiating market area based on the members' clinical services at the early stage of network development.

There was more involvement in marketing efforts in clinic-hospital relationships than in clinic-clinic relationships. Generally speaking, hospitals have more resources (i.e., money, human resources, materials, and physical assets) than clinics, which might explain the stronger marketing involvements between the clinic and hospital members, including the library sharing (books and literatures), facility brochure dissemination, public promoting, and medical research cooperation. These integration efforts also meet the expectation of the national health authority for resource sharing and medical quality image enhancement among the health care providers.

Financial infrastructure was found to be the least integrated, with most items never considered. Perhaps the only reason for the higher score of budget planning activities (Table 5: items 58 and 77) was that BHNI required each PCCN to design and determine its budgeting arrangement in advance before joining the demonstration project. While slightly more financial involvement was made among network members (Table 5: items 68, 72 & 73), possibly due to similar needs, there remains a lot of room for financial integration in the future.

There was a need for networks to develop electronic information systems, though creating and managing an integrated information system involves very detailed work. Most of the clinics surveyed have focused more on the individual public members' administrative works such as filing patient medical records, collecting and managing network patient clinical data, and scheduling clinical services, which were required by the BNHI. The factors for the health care managers to adopt the integrated clinical information systems include the decision of make or buy, adoption leadership, adoption objectives, implementation leadership, phased versus simultaneous implementation, parallel systems, information technology implementation policies and practices, use levels and resistance, and realized benefits and return on investment calculation [66], which might be very cumbersome and time-consuming. It suggests that the network partners might be engaged, firstly, more in simpler network cooperation such as the administrative systems for patient admission to the network members and establishing united web pages for patients to access their family physicians and network members for medical and public promotion purposes. And for further integrated information investments, efforts must be redirected for network members to work together to define the approach to specific classes of integration for the long term [74].

## Conclusion

This study tried to portray and trace how the facility participants were involved in the Taiwan PCCNs. It was found that Taiwan PCCNs' members had higher involvement in the governance infrastructure, which was usually viewed as the most important for establishment of core values in PCCNs' organization design and management. There existed a higher extent of integration of clinical, marketing, and information infrastructures among the hospital-clinic member relationship than those among clinic members within individual PCCNs. The financial infrastructure was shown the least integrated relative to other functional infrastructures at the early stage of PCCN formation. Page [43] argued that networks form and grow for various reasons, however, only some of them could be compatible with the iterative processes of collaboration. Some participants in the PCCNs may simply seek short-term economic gains and have little interest in joint learning and continuous improvement. From an organizational design perspective, the old phrase proposed by the wisdom of the saying about developing the integrated organizations (networks) should be – "coming together is the beginning, and working together is the success." Page [43] examined the virtual provider organizations such as physician-hospital organizations and pointed out the issue of the provider attitudes and behaviors as the critically successful continuous improvements in the health care environments. A wide variance of degree of network integration in Taiwan PCCNs still leaves room to improve.

In this study, the thoroughly surveyed items, that is, the potential network design content, were employed. In addition to provide how the network members have done their initial work at the early stage of network forming in this study, the detailed surveyed items, the concepts proposed by the managerial and theoretical professionals, could be also a guide for those health care providers who have a willingness to join multi-organizations. It suggests that health care providers could take more detailed looks about those surveyed items and give some possible opportunities to create the potential actions. Further research could be empirically done to explore the relative influence of these integration mechanisms on the effectiveness of organizational partnerships.

The partnerships within each PCCN represent various relationships that depend on how much the members are engaged in the projects. In addition to the macro concepts including governance, clinical, marketing, financial, and information infrastructures explored in this study, other managerial issues for integrated organizations were also suggested such as formation of an integrated cultural atmosphere, human resources management, physician involvement, mission and commitment establishment, from micro organizational behavior perspective [30-32,34,36,75,76]. Micro managerial and longitudinal research designs could be employed to more precisely catch the never completing integration efforts in the future.

## Abbreviations

primary community care network (PCCN); Bureau of National Health Insurance (BNHI)

## Competing interests

The author(s) declare that they have no competing interests.

## Authors' contributions

BYJL independently designed and conducted this study.

## Pre-publication history

The pre-publication history for this paper can be accessed here:


